# Universal or Specific? A Modeling-Based Comparison of Broad-Spectrum Influenza Vaccines against Conventional, Strain-Matched Vaccines

**DOI:** 10.1371/journal.pcbi.1005204

**Published:** 2016-12-15

**Authors:** Rahul Subramanian, Andrea L. Graham, Bryan T. Grenfell, Nimalan Arinaminpathy

**Affiliations:** 1 Department of Ecology and Evolution, University of Chicago, Chicago, Illinois, United States of America; 2 Department of Ecology and Evolutionary Biology, Princeton University, Princeton, New Jersey, United States of America; 3 Department of Infectious Disease Epidemiology, Imperial College London, London, United Kingdom; ETH Zurich, SWITZERLAND

## Abstract

Despite the availability of vaccines, influenza remains a major public health challenge. A key reason is the virus capacity for immune escape: ongoing evolution allows the continual circulation of seasonal influenza, while novel influenza viruses invade the human population to cause a pandemic every few decades. Current vaccines have to be updated continually to keep up to date with this antigenic change, but emerging ‘universal’ vaccines—targeting more conserved components of the influenza virus—offer the potential to act across all influenza A strains and subtypes. Influenza vaccination programmes around the world are steadily increasing in their population coverage. In future, how might intensive, routine immunization with novel vaccines compare against similar mass programmes utilizing conventional vaccines? Specifically, how might novel and conventional vaccines compare, in terms of cumulative incidence and rates of antigenic evolution of seasonal influenza? What are their potential implications for the impact of pandemic emergence? Here we present a new mathematical model, capturing both transmission dynamics and antigenic evolution of influenza in a simple framework, to explore these questions. We find that, even when matched by per-dose efficacy, universal vaccines could dampen population-level transmission over several seasons to a greater extent than conventional vaccines. Moreover, by lowering opportunities for cross-protective immunity in the population, conventional vaccines could allow the increased spread of a novel pandemic strain. Conversely, universal vaccines could mitigate both seasonal and pandemic spread. However, where it is not possible to maintain annual, intensive vaccination coverage, the duration and breadth of immunity raised by universal vaccines are critical determinants of their performance relative to conventional vaccines. In future, conventional and novel vaccines are likely to play complementary roles in vaccination strategies against influenza: in this context, our results suggest important characteristics to monitor during the clinical development of emerging vaccine technologies.

## Author Summary

Influenza vaccines used today offer good protection, but have limitations: they have to be updated regularly, to remain effective in the face of ongoing virus evolution, and they cannot be used in advance of an influenza pandemic. In this study we considered how such ‘conventional’ vaccines might compare on the population level against new ‘universal’ vaccines currently being developed, that may protect against a broad spectrum of influenza viruses. We developed a mathematical model to capture the interactions between vaccination, influenza transmission, and viral evolution. The model suggests that annual vaccination with universal vaccines could control annual influenza epidemics more efficiently than conventional vaccines. In doing so they could slow viral evolution, rather than promoting it, while maintaining the broadly protective immunity that could mitigate against the emergence of a pandemic. These effects depend sensitively on the duration of protection that universal vaccines can afford, an important quantity to monitor in their development. In future, it is likely that conventional and universal vaccines would be deployed in tandem: we suggest that they could fulfill distinct roles, with universal vaccines being prioritised for managing transmission and evolution, and conventional vaccines being focused on protecting specific risk groups.

## Introduction

Seasonal and pandemic influenza pose major public health challenges [1, 2] Vaccines against seasonal influenza aim to raise antibodies against the hemagglutinin (HA) and neuraminidase (NA) surface proteins of circulating strains [3]. While these targets offer the best correlates for immune protection, they are also by far the most variable amongst influenza viral components [4, 5], undergoing continual evolution for immune escape: current seasonal influenza vaccines therefore need to be updated regularly. Moreover, influenza pandemics are caused by the emergence of a virus with an altogether new HA (and other viral components), to which there is little or no immunity in the human population [6, 7]. Current vaccines cannot be deployed in advance of an influenza pandemic, as it is not possible to predict what virus will cause the next pandemic [8].

There is evidence to suggest that other viral components, including the matrix protein M1 and the nucleoprotein NP, may be more conserved than HA and NA [9–13]. Immunity to these proteins, mediated by T-cells rather than by antibodies, is associated with broad-spectrum protection[14], even against novel pandemic viruses [15, 16]. At the same time, it is also possible for antibodies to raise broad-spectrum protection: with most of HA variability concentrated in the ‘head’ region of the protein, antibodies against the more conserved (but less accessible) ‘stem’ region have also attracted considerable attention[17–19]. Antibodies against the ion channel protein M2 have also been shown to elicit broad protection [20, 21].

Only with recent advances in vaccine technology has it been possible to target these alternative viral components [10, 17–19, 22–24]. The resulting emergence of candidates for ‘universal’ vaccines raises the potential for more stable influenza vaccination programmes, that do not have to be updated so frequently. At the same time, even with current, strain-matched vaccines, population coverage is on an increasing trend: in some settings (notably in the UK) there is growing emphasis on widened vaccination coverage to reduce transmission as well as disease [25]. Coverage in the US has been steadily rising and has recently exceeded 43% of the population [26]. These trends suggest that annual, mass influenza immunization programmes could foreseeably become a reality.

Together, such developments raise important questions about the potential future use and impact of influenza vaccines. For example, how might novel vaccines compare against current, strain-matched vaccines, in their ability to control transmission? What are the implications for seasonal HA evolution, of a mass immunisation programme targeting HA versus one targeting other more conserved viral components? As these vaccines are still in development, important vaccine parameters, including classical vaccine efficacy, and duration of protection in humans, remain to be determined [27]: what are the implications of these vaccine characteristics, for future immunization programmes?

Previous work [28] focused on the emergence of a pandemic virus, finding that the ability of cross-protective vaccines to mitigate pandemic risk depended on the ability of any vaccine (whether current or future) to provide broader protection than that provided by natural infection. Another modeling study [29] showed how cross-protective vaccines could slow the rate of antigenic evolution for seasonal viruses, thus enhancing the control of seasonal epidemics with conventional (HA-specific) vaccines. However, neither model addressed the potential effect of conventional vaccines on seasonal viral evolution, and how this might compare with universal vaccines. The present model builds on this previous work, addressing the questions above with a simple, novel model of influenza transmission and evolution. The model evaluates the relative merits of ‘conventional’ versus ‘universal’ vaccination, while casting light on vaccine characteristics that would be helpful to quantify, in anticipation of novel vaccine candidates entering advanced clinical trials.

## Methods

To motivate the model, we first give a brief overview of influenza vaccines. At present one of the most widely used influenza vaccines is the trivalent inactivated vaccine (TIV) [3]. Consisting only of non-replicating viral material, this formulation raises antibodies against HA and NA, but no T-cell immunity. Another formulation is the live-attenuated influenza vaccine (LAIV), using cold-adapted influenza viruses to target specific strains of HA and NA [30] Although such vaccines raise T-cell immunity through viral replication, a recent study suggests that they offer only modest efficacy against antigenically drifted strains [31] and reduced heterosubtypic immunity [32], comparable to that of TIV. Nonetheless, cross-protection could be enhanced through adjuvants or T-cell boosting [33, 34].

Meanwhile, emerging candidates for ‘universal’ vaccines focus on the exclusive expression of cross-protective immunogens, whether T-cell targets [23, 24] or the conserved HA stem [17, 19, 35]. Such vaccines have shown protection against heterosubtypic challenge in animal models [19, 24, 35], as well as in human challenge studies [36]. A limitation amongst T-cell vaccines in particular is that—unlike immunity to HA or NA—they do not block infection, but rather control the severity of disease [37]. Nonetheless, in doing so, they substantially reduce the amount and duration of viral shedding, thus reducing opportunities for transmission [24, 36].

In this context, we concentrate on the potential, future impact of mass vaccination programmes. We distinguish two types of immunity in the model: ‘strain-specific’ immunity is long-lasting and blocks infection against a given (immunizing) HA strain, and offers some protection against related strains, diminishing with antigenic distance from the immunizing strain. ‘Cross-protective’ immunity—consistent with T-cells—wanes over time [38, 39] but acts uniformly against all HA strains: conservatively, we assume that this type of immunity does not reduce susceptibility, but instead lowers infectiousness in the event of infection.

We assume that natural infection raises both types of immunity: further, we assume that ‘conventional’ vaccines (TIV and LAIV) raise only effective strain-specific immunity, and that ‘universal’ vaccines (concentrating here on T-cell vaccine candidates) raise only effective T-cell immunity. In the current work, this dichotomous choice of vaccine effect helps to contrast the corresponding population-level effects that arise. In practice, however, a ‘cocktail’ vaccine formulation could combine both types of vaccine effect: although beyond the scope of this paper, we discuss potential implications of this type of vaccine below. For a summary of the immune transitions in the host population, see Fig A in S1 Appendix.

The model has two main, coupled components: The ‘epidemic component’ captures the acquisition of immunity through vaccination and natural infection, while the ‘interepidemic component’ captures the loss of immunity in the population through antigenic drift; waning of cross-protective immunity; and population turnover. We describe these both in turn.

### 1. Epidemic component

**Fig 1:**
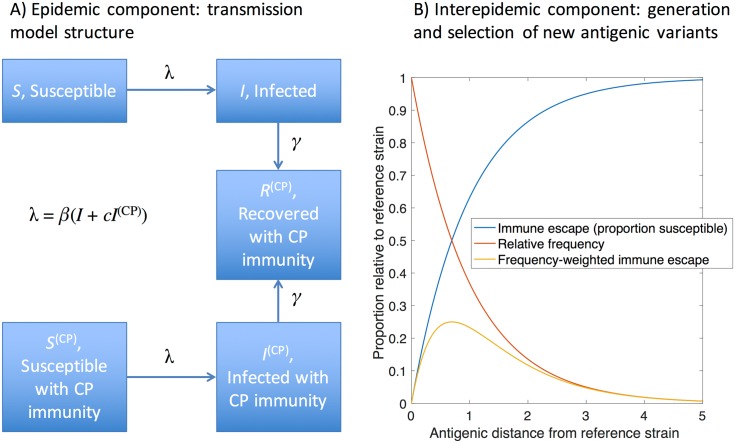
Overview of the two major model components. (A) The epidemic component, a deterministic, compartmental model capturing a single influenza season. Boxes represent proportions of the population in different states, where ‘CP’ denotes cross-protective immunity. Here we assume conservatively that the role of cross-protective immunity is to reduce infectiousness without necessarily reducing susceptibility: as a result in the force-of-infection term *λ*, there is a coefficient *c* < 1 to represent diminished infectiousness amongst *I*^*(CP)*^. (B) The ‘interepidemic’ component, which governs the generation and selection of new strains. Given a reference, immunizing strain at *d* = 0, we assume that candidate viruses at increasing antigenic distance (horizontal *x*-axis) show increasing immune escape (blue curve). However, these candidates are also assumed to be less frequent during the interepidemic period (red curve). The selected virus is assumed simply to be that which maximises the trade-off between these factors (yellow curve). In the full model, this is calculated with respect to the different strains that individuals in the population have last been infected with, as described in the main text and in the appendix.

The epidemic component is a deterministic, compartmental framework that models each seasonal epidemic as a single epidemic wave, with a single circulating strain (Fig 1A). For simplicity we ignore age structure, as well as spatial heterogeneities, assuming simply a fully ‘well-mixed’ population. The governing equations are as follows:

$$\begin{array}{cc} \frac{dS}{dt}=-\lambda S & \frac{dI}{dt}=\lambda S-\gamma I\\ \frac{dS^{\left(cp\right)}}{dt}=-\lambda S^{\left(cp\right)} & \;\;\;\;\;\;\;\frac{dI^{\left(cp\right)}}{dt}=\lambda S^{\left(cp\right)}-\gamma I^{\left(cp\right)}\\ \;\;\;\;\;\;\;\frac{dR^{\left(cp\right)}}{dt}=\gamma \left(I+I^{\left(cp\right)}\right) & \end{array}$$ (1)

Here *S*, *I*, *R* are respectively the proportions of the population who are susceptible to infection; infectious; and recovered and immune. The superscript (*cp*) marks individuals having cross-protective immunity (but not strain-specific immunity); *γ* is the per-capita rate of recovery; and *λ* is the force of infection, given by:

$$\lambda =\beta I+\left(1-c\right)\beta I^{\left(cp\right)}$$

Here *β* is the effective contact rate, multiplied by the average number of infections per infected case, and *c* is the reduction in infectiousness arising from cross-protective immunity, written so that *c* = 1 corresponds to fully transmission-blocking immunity.

The initial conditions are given by the proportion of the population that has HA-specific immunity, given prior epidemic sizes and the amount of antigenic drift that has occurred (see below), along with two types of vaccination programme, which are completed prior to each epidemic and with random coverage, irrespective of an individual’s exposure history or immune status: conventional vaccination displaces individuals from *S* to *R* and from *S*^*(cp)*^ to *R*^*(cp)*^, while universal vaccination displaces individuals from *S* to *S*^*(cp)*^ and *R* to *R*^*(cp)*^.

For comparability between the two types of vaccine being considered here, it is necessary to choose values for the quality of vaccine protection (vaccine ‘efficacy’) that are matched in terms of their population effect. We assume for simplicity that universal vaccination elicits the same cross-protective immunity as does natural infection, thus identifying *c* with the efficacy of universal vaccination. Correspondingly for conventional vaccines, we assume that efficacy derives from a proportion *c* of vaccinated individuals successfully acquiring strain-specific immunity (the rest remaining with their prior immune status). It is straightforward to show (see appendix) that both vaccines thus have the same effect on *R*_0_.

The initial conditions are given by the proportion of the population that has HA-specific immunity, given prior epidemic sizes and the amount of antigenic drift that has since occurred (see below), along with two types of vaccination programme, which are completed prior to each epidemic and with random coverage: conventional vaccination displaces individuals from *S* to *R* and from *S*^(cp)^ to *R*^(cp)^, while universal vaccination displaces individuals from *S* to *S*^*(cp)*^ and *R* to *R*^*(cp)*^.

### 2. Interepidemic component

In the interepidemic period, we model a loss of immunity in the population due to three mechanisms: loss of strain-specific immunity through antigenic drift, loss of cross-protective immunity through waning of T-cell immunity, and a general depletion of immunity through population turnover (replacement of immune hosts by susceptible ones). These are implemented as follows.

For antigenic drift we adopt a simple deterministic framework to capture the essential role of population immunity, in driving selection for new variants (see, for example, ref [40] for a review of evidence supporting this assumption). We assume a one-dimensional axis of HA antigenic variation, a simplified representation of the distinctive ladder-like phylogeny of influenza A hemagglutinin [41]. Fig 1B shows the simple case of a single immunizing strain (a ‘reference’ strain). We denote *d* as the antigenic ‘distance’ between this and a candidate virus, shown on the horizontal axis. The Figure captures two essential features of antigenic evolution: first, candidates with greater *d* have a greater degree of immune escape and therefore a higher transmission potential [39] (blue curve). However, they arise at a lower frequency (red curve). Combining these two opposing factors to yield the ‘frequency-weighted immune escape’ (orange curve), we assume that—on a population level—the selected virus for an upcoming season is one that maximizes this quantity. Specifically, the frequency-weighted immune escape for this candidate virus is defined as:

F(d)=exp(−kd)[1−exp(−d)], (2)

where *k* is a parameter governing the relative rarity of immune escape variants. For example, in the theoretical case *k* = 0 there is unlimited viral diversity in the interepidemic period, thus allowing a pandemic-scale outbreak every year. At the other extreme as *k* → ∞, there is no generation of escape variants even in the face of population immunity: a situation similar to measles. For influenza, the scenario is intermediate. We calibrate the value of *k* in order to yield, at steady state, seasonal epidemics that infect roughly 10% of the population per season, consistent with the behaviour of seasonal influenza [42–44].

While eq (2) is in the simple case of a population with exposure to only one virus, over several seasons there is a series of viruses that emerge and circulate. Moreover, conventional vaccination in any given season offers protection against the virus circulating in that season, but also—to an extent diminishing with antigenic distance—against related viruses. It is thus necessary to keep track of the exposures to these viruses in the population, and to evaluate the proportion susceptible over all of these histories. Nonetheless, as we assume a one-dimensional antigenic space, it is only necessary to record the most recent infection or vaccination that individuals have undergone. Details of the necessary record-keeping are provided in the appendix.

For the waning of T-cell immunity, we assume simply that a proportion *σ* of individuals lose this immunity in every interepidemic period. For illustration we choose *σ* = 0.21, consistent with findings from early seminal work that suggested a T-cell half-life of 3 years [38]. However, it is important to note that there is considerable uncertainty around this Figure, with more recent studies suggesting that CD8 T-cell immunity can last as long as a decade, both for influenza ([45]) and for other viruses ([46]). Accordingly, we explore this uncertainty in the work below.

**Fig 2:**
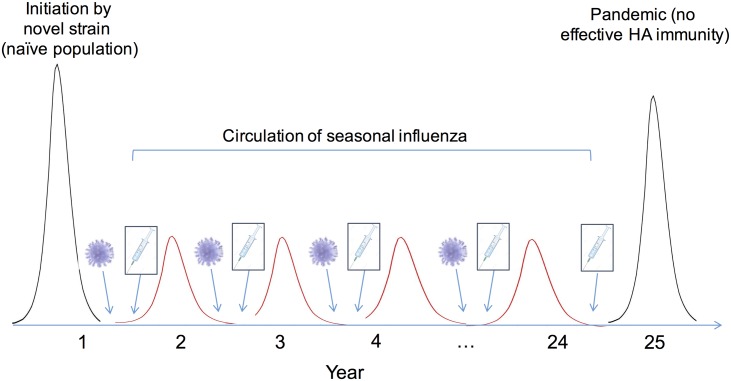
Schematic illustration of the simulation sequence. As described in the Methods, the simulation is initiated by a pandemic in a naïve population. In subsequent seasons we assume that strain selection happens during the interepidemic period (annotated by a virus in the Figure, and corresponding to Fig 1B). This leads to a loss of strain-specific immunity due to antigenic drift, and accompanies a loss of immunity through population turnover, as well as through decay of cross-protective immunity. We assume that routine vaccination, whether conventional or universal, occurs just prior to each seasonal epidemic (annotated by a syringe in the Figure). The epidemic that follows is governed by the eq (1) in the main text, leading to a gain of immunity in the population (corresponding to Fig 1A)). We iterate through seasons in this way, ultimately reporting the ‘seasonal epidemic size’ as the mean epidemic size between seasons 5 and 24, and the ‘pace of antigenic evolution’ as the mean distance between successive strains during this period. Finally, we simulate a pandemic in year 25, assuming a virus to which cross-protective immunity, and not strain-matched immunity, is effective.

Table 1 shows the default parameter values used, and Fig 2 schematically summarises the procedure. Starting with a virus in a fully susceptible population, we simulate its spread using (1) (‘initiation’ in Fig 2). We then simulate the selection for a new immune escape variant using (2). Having determined this variant, we find the associated initial conditions (population susceptibility) for the subsequent epidemic season, and repeat the iteration from (1) to (2) (‘Circulation’ in Fig 2). Finally, to study how a pandemic would be affected by the conditions of immunity in this population, at year 25 we introduce a virus to which only pre-existing cross-protective immunity, and not HA-immunity, is effective (‘Pandemic’ in Fig 2).

**Table 1:** Parameter values used in the model. ‘Default values’ are used in Fig 3, while parameter ‘ranges’ are used in Figs 4 and 5. Notes: a) For comparability between the two types of vaccines, we choose *ϵ* = *c*. b) *R*_0_ is given by the ratio *β/γ* in the model: their individual values do not independently affect epidemic sizes, so it is only necessary to choose *R*_0_. c) Value corresponds to a mean lifetime of 70 years. d) Under the baseline values shown here, *k* is tuned to give seasonal epidemics infecting roughly 10% of the population. e) In the absence of a natural scale for *k*, we simply take half and twice the baseline value.

Parameter	Description	Default value	Range
***c***	Reduction in infectiousness for individuals arising from cross-protective immunity (*c* = 1 represents fully transmission-blocking immunity).	0.65	0.5–0.8
***ϵ***	Efficacy of conventional vaccination, modelled as proportion of vaccinated individuals successfully acquiring strain-specific immunity	0.65^a^	Matched with *c*
***h***	Half-life of cross-protective immunity (years)	3	0.25–10 (i.e. 4 months to 10 years)
***σ***	Waning rate, proportion of individuals losing cross-protective immunity annually	0.21	Governed by *h*, calculated as $\sigma =\left(1-0.5^{\frac{1}{h}}\right)$
***R***_**0**_	Basic reproduction number	2.0^b^	1.1–3
***μ***	Mean mortality rate	0.014^c^	0.011–0.02
***k***	Constant governing the abundance of immune escape variants, relative to their immune escape potential (see Fig 1B)	0.34^d^	0.15–0.6^e^

Although showing a steady state in Fig 2, there are certain conditions where simulated seasonal influenza epidemics can show minor annual variations, as described below. Accordingly, we measure the ‘seasonal epidemic size’ as the mean epidemic size from years 5 to 24. We additionally define the ‘pandemic size’ as the size of the pandemic when introduced at year 25.

### 3. Sensitivity analysis

The default parameter values shown in Table 1 (second column) are helpful for illustrating model behaviour. To examine the robustness of our model results to variation in these parameters, we then simulate the model through the range of plausible parameter values shown in Table 1 (third column). In particular, using latin hypercube sampling, we generate 10,000 parameter sets within the ranges shown. To ensure plausible epidemiology, we retain those parameter combinations yielding seasonal epidemics that infect between 5 and 20% of the population, consistent with estimates that influenza infects roughly 10% of the population each season [45–47]. Under this parameter set, we then investigate the variability in the relative performance of conventional vs universal vaccines.

## Results

**Fig 3:**
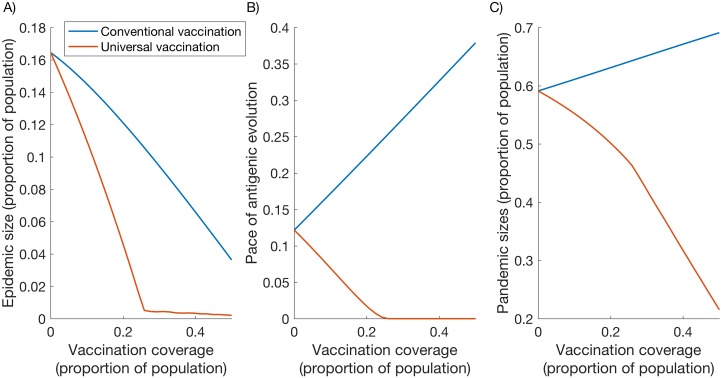
Comparative vaccine effects under illustrative parameters. The Figure compares universal vaccines (orange) against conventional (blue), under different levels of coverage in the population. (A) The proportion of the population being infected by seasonal influenza, each year (B) The ‘pace’ of antigenic change, measured by the mean antigenic distance between successive seasons. (C) The size of a pandemic following several years of seasonal vaccination. In all Figures, cross-protective immunity is assumed to have a half-life of 3 years, and both HA-specific and universal vaccination occur annually. See Table 1 for parameter values.

Fig 3 provides a side-by-side comparison of the effects of conventional and universal vaccines, presenting three different outcomes: control of seasonal epidemics (panel A); the effect of vaccines on the pace of antigenic evolution (panel B); and the implications of seasonal vaccination for pandemic sizes (panel C). As described above, the Figure assumes equivalent vaccine efficacy and, in both cases, an annual vaccination program. The Figure is illustrative, involving only the point estimates for each of the input parameters involved (Table 1): below we examine the robustness of this qualitative behaviour under parameter variability.

First, Fig 3A illustrates how conventional and universal vaccines could have differing effects on long-term patterns of influenza transmission. While both vaccines reduce seasonal epidemic sizes, at any given level of coverage, universal vaccines appear to have a stronger effect in suppressing seasonal epidemics. Moreover, Fig 3B illustrates—consistently with previous work—that large-scale universal vaccination would slow antigenic evolution over several seasons. Notably, however, these results suggest that conventional vaccines would tend to do the opposite, potentially accelerating antigenic change. We discuss below how these effects might arise from the different types of vaccine action.

Under universal vaccination, the pace of antigenic evolution is driven to zero at sufficiently high coverage (Fig 3B, orange curve): in this regime seasonal transmission is so heavily dampened that there is little strain-specific immunity to drive selection for new variants. However, we note that seasonal epidemics—even of very small sizes—could still occur at this coverage (Fig 3A, ‘elbow’ in orange curve). This is a regime where universal vaccines interrupt transmission in the short term: over several years, however, seasonal viruses can sporadically persist, purely because of the accumulation of naïve individuals, rather than because of antigenic evolution—a situation analogous to measles ([47]) but with a substantially lower *R*_*0*_. As discussed below, however, spatial and stochastic dynamics would greatly affect these extreme cases.

Fig 3C additionally illustrates differences between the vaccines, for the size of a pandemic following several years of seasonal vaccination. The Figure illustrates that, while both types of vaccines can reduce seasonal epidemic sizes, high vaccination coverage with conventional vaccines tends to allow for increased pandemic sizes, whereas universal vaccines have the opposite effect. Moreover, pandemic sizes decline more rapidly with increasing universal vaccination coverage when there is no antigenic evolution (i.e. an increased gradient in pandemic sizes for vaccine coverage > 25%). This effect arises because interrupting transmission renders vaccination the sole source of cross-protective immunity in the population. The incremental impact of increased vaccination coverage is thus greater than in regimes allowing transmission, where infection is an additional source of cross-protective immunity.

To additionally explore the validity of these findings under parameter uncertainty, we conduct a multivariate sensitivity analysis as described in the Methods. In particular, we explore the key outputs of this analysis: the relative performance of conventional and universal vaccines, with respect to control of seasonal influenza; impact on the pace of antigenic evolution; and implications for pandemic control. Taking the first of these as an example, if *g_C_* is the average seasonal epidemic size under a given vaccination coverage, and *g*_*U*_ is the corresponding quantity for a universal vaccine, we calculate the ratio *r* = *g*_*U*_ / *g*_*C*._ As long as this quantity is below 1, the qualitative finding in Fig 3A holds true. Defining *r* as the ‘relative efficiency’ in control of seasonal epidemics, we likewise consider relative efficiencies in controlling antigenic evolution, and in pandemic control (corresponding to each of the panels in Fig 3).

**Fig 4:**
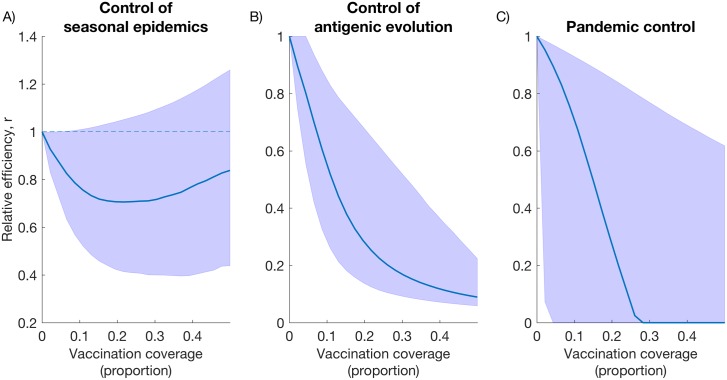
Relative performance of conventional and universal vaccines, under different levels of vaccination coverage. Shaded areas represent the range of outcomes arising from the parameter ranges shown in Table 1: lower and upper boundaries depict the 2.5^th^ and 97.5^th^ percentiles of simulated outcomes, respectively. In each panel. where ratios are greater than 1, conventional vaccines have greater efficiency than universal vaccines, and vice versa. (A) Relative performance in controlling seasonal epidemics, defined as the ratio of seasonal epidemic sizes under a given coverage of vaccination, comparing conventional and universal vaccines (i.e. upper and lower curves in Fig 3A). (B) Relative performance in controlling antigenic evolution, defined as the ratio in the pace of antigenic evolution, comparing conventional with universal vaccines (i.e. upper and lower curves in Fig 3B). (C) Relative performance in controlling pandemics, defined as the ratio of pandemic sizes, comparing conventional with universal vaccines (i.e. upper and lower curves in Fig 3C).

Fig 4 plots these relative efficiencies, together with their uncertainty, for different levels of vaccination coverage. In each panel, the region above the dashed line (i.e. a ratio > 1) corresponds to conventional vaccines being more efficient than universal vaccines, and vice versa. Fig 4B and 4C suggest that universal vaccines are robustly more efficient in controlling antigenic evolution and in mitigating pandemic sizes. Notably, however, the uncertainty bounds in Fig 4A straddle the line *r* = 1 (shown ‘dashed’), indicating certain parameter combinations under which a universal vaccine could allow *greater* seasonal epidemics than a conventional vaccine.

**Fig 5:**
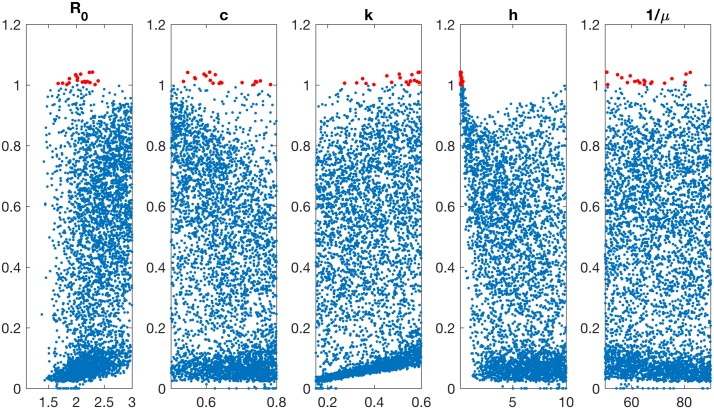
Sensitivity analysis of relative efficiency in controlling seasonal epidemics. Relates to Fig 4A, and assuming 15% coverage. The quantity *r* is the relative seasonal epidemic size under 15% coverage of a universal vaccine, relative to that under the same coverage of a conventional vaccine. Points with *r* > 1 (shown in red) universal vaccines are less efficient than conventional vaccines, and vice versa. Each panel shows results over 10,000 simulations spanning the parameter ranges in Table 1 (third column), as a scatter plot with respect to each of the parameters in the model. Note here that each point has the same height in each panel; they are simply arranged in different ways along the horizontal axis, depending on their relationship to the model parameter denoted on that axis. The median and 95% intervals for *r* are 0.38 (0.03–0.92). Parameters are as follows: *R*_*0*_, basic reproduction number; *c*, effect of cross-protective immunity in reducing transmission potential; *k*, abundance of immune escape variants, relative to immune escape potential; *h*, half-life of cross-protective immunity; 1/*μ*, mean host lifetime.

To identify which parameters are driving this result, taking a vaccination coverage of 15%, Fig 5 shows scatter plots of the relative efficiency r with respect to each of the parameters in the model. Points of interest (*r* > 1) are shown in red, and are roughly evenly distributed for each of the parameters, with the notable exception of *h* (fourth panel), where values of *r* > 1 clearly cluster around a low duration of protection. Motivated by this Figure, holding *h* constant at its default value, and re-sampling other parameters, yields values of *r* strictly less than 1 (see Fig C in S1 Appendix). Overall, therefore, in the range of parameter values explored here, universal vaccines appear robustly more efficient in controlling seasonal epidemics, as long as the duration of protection that they provide is sufficiently long.

## Discussion

While a major focus in the development of new influenza vaccines is on their ability to provide individual protection, anticipating the population-level effects of vaccination can also yield useful public health insights. Here, we present a simple model bringing together influenza evolution and epidemiology, and use this model to compare vaccination programmes with two different types of influenza vaccine: current, ‘conventional’ strain-matched vaccines, versus emerging, ‘universal’ vaccines.

A primary result from this work is the contrasting evolutionary effects associated with the two types of vaccines. In general, sustained control of transmission reduces the number of immune individuals in the population, and thus dampens selection pressure for new antigenic variants (Fig 3B): universal vaccines, because they are not HA-specific, are able to achieve this state without themselves contributing to HA selection pressure. Thus the pace of antigenic evolution decreases with higher universal vaccine coverage. However, conventional vaccines raise strain-matched immunity: they therefore have the opposite effect to universal vaccines, compounding HA selection pressure and thus tending to accelerate antigenic evolution.

In control of seasonal influenza, universal vaccines could also avert more transmission per dose administered than conventional vaccines, with the potential to interrupt transmission even at moderate levels of coverage (Fig 3A). This amplified effect likely arises from the fact that universal vaccination reduces strain-matched immunity in the population while increasing cross-protective immunity and slowing antigenic drift, while conventional HA-specific vaccines do the converse. Overall, therefore, universal vaccination could complement population immunity in a way that is more efficient for controlling transmission, over several seasons, than strain-matched vaccines. Furthermore, while the effects of HA-specific vaccination are limited by antigenic evolution, the effects of universal vaccination are limited by the duration of cross-protective immunity [28]. As long as this duration is long enough to persist across vaccination intervals, the effect of cross-protective vaccination can be maintained on a population level with each passing season.

Conventional vaccines provide HA-specific immunity against seasonally circulating strains at the expense of infection-acquired immunity that may otherwise protect against novel antigenic subtypes [48–50]. Thus, as shown in Fig 3C, increased HA-specific vaccination coverage could result in increased pandemic sizes. Indeed, these model findings are consistent with experimental findings in animal challenge studies [50]. By contrast, a universal vaccine, even if transmission-blocking rather than infection-blocking, could reduce pandemic sizes by promoting cross-protective immunity in the population. Similar phenomena have been suggested by Zhang et al via different mechanisms, by which cross-protective immunity limits the opportunities for reassortment, thus limiting the emergence of pandemic-capable viruses ([28]). Taken together, these findings suggest that universal vaccines could be effective in both preventing and mitigating pandemic emergence.

While influenza is a readily evolving pathogen, it is evidently not so rapidly evolving as to cause pandemic-scale epidemics every season. Here, we capture this phenomenon by assuming that viral evolution is limited by the available HA diversity in the population (Fig 1B). As for the conserved antigens targeted by universal vaccines, we have ignored the potential for immune escape, assuming in this work that any antigenic change would be too functionally costly for the virus to continue replicating. Nonetheless, the potential for such immune escape cannot be discounted: should it occur it would have far reaching consequences, comparable to pandemic emergence. Additionally, even if conventional vaccines should have negative implications for pandemic control, for their sterilizing immunity they would remain essential in routine immunization to protect specific risk groups such as the immunocompromised and the elderly.

Overall then, rather than replacing one vaccination programme with another, it is important to consider universal vaccines as being strategically complementary to conventional, strain-matched vaccines. With recent work highlighting the potential effects of influenza vaccination programs in controlling transmission [25], our work suggests that—depending on the characteristics of new vaccines including duration of protection and vaccination frequency—the ‘transmission dampening’ role could be one best filled by universal vaccines. An alternative could be a ‘cocktail’ formulation consisting of a combination of strain-specific and vectored, cross-protective immunogens. Such cocktails could continue to protect clinical risk groups such as the elderly, as well as maintaining cross-protective immunity in the population to mitigate pandemic risk. However, their effect on seasonal influenza evolution would depend on the relative strengths of strain-specific and cross-protective protection that they provide: future work could explore the extent to which the cross-protective component of a cocktail vaccine could mitigate the potential ‘evolution-speeding’ effects of its strain-specific component.

The present model has several limitations to note. First, it involves a stylized model of influenza evolution: in practice, the antigenic dynamics of influenza arise from a combination of complex processes, spanning the chance emergence of an immune escape variant in an infected host; the transmission of that mutant to other hosts; and its successful establishment in the global population, all in competition with other potential escape variants ([51, 52]). Each of these stages is stochastic, giving rise to notable irregularities in influenza evolution such as antigenic ‘jumps’ shown by influenza A, every 3–9 years, with important consequences for vaccine selection [41] There is also notable variation in the geographical source of circulating influenza strains each year. [53] Nonetheless, the aim of the present work is not to explain such spatiotemporal variation, but rather to capture the essential, long-term interplay between population immunity and viral evolution. Consequently our current findings for universal vaccines (particularly, that they could slow antigenic evolution) are consistent with previous work, which employed a more complex, stochastic framework [29, 54]: we would expect our current findings for conventional vaccines to be similarly qualitatively robust to stochasticity in viral evolution.

Second, the model does not take into account heterogeneities such as age structure [45,46]. With school-age children playing an important role in the transmission of influenza ([55–57]), and the elderly being less important for transmission, the effect of a given population coverage of vaccination will depend critically on how it is distributed amongst age groups ([58]). Neglecting such effects, our model may overestimate the impact of a given vaccination coverage, for example, suggesting such low seasonal epidemic sizes at current levels of coverage in the US (Fig 3A). If this bias applies equally for universal as well as for conventional vaccines, it may not be expected to influence our overall results about their relative efficiencies. Moreover, an important area for future work would be the potential impact of age-targeted vaccination programmes for emerging, transmission-controlling vaccines.

Several important caveats about immunity also bear mention: first, in the absence of relevant data, we have assumed that vaccine-induced immunity has an efficacy equivalent to its counterpart in natural immunity. Further work could explore the implications of relaxing this assumption. It might be expected that model results would depend to a large extent on whether vaccine-induced immunity would be more or less effective (or long-lasting) than its counterpart in natural immunity. (It is notable, for example, that recombinant technology raises the prospect of focusing immunity on particular antigens to a greater extent than is possible through natural immunity [22, 59]). Second, for simplicity we have neglected the potential for complex interactions such as between antibody-mediated and T-cell mediated immunity, and the potential effect of an individual’s infection history on their vaccine response [60–64]. These complexities are only starting to be explored for influenza, and in future a better understanding of these immunological interactions will allow refined models to explore their implications. Third, we have assumed that universal vaccination does not protect against infection (i.e. no reduction in susceptibility). This being a conservative assumption, we might expect our overall findings to be accentuated by allowing for such additional protection. Conversely, we have assumed that current, strain-matched vaccines elicit no cross-protective immunity. Although this is a helpful caricature for contrasting two different modes of vaccination, conventional vaccines may also elicit some heterosubtypic immunity [65]. In practice, any such protection is unfortunately too weak for current vaccines to protect against novel pandemic strains [32], a major rationale for universal vaccines [23, 35]–nonetheless, any broad protection from current vaccines would tend to narrow the gap between conventional and universal vaccines illustrated in Fig 3. Such caveats notwithstanding, our overall findings are likely to hold true: a vaccine formulation enhancing cross-protective over strain-specific immunity would have qualitatively different population implications from one that does the converse. Overall, a key data need in future is a quantitative comparison of the duration and potency of cross-protection raised by current vaccines, against that offered by emerging vaccine candidates.

In summary, emerging vaccine technology, along with increasing interest in understanding the biology of influenza evolution, are offering fresh prospects for the control of influenza. In the context of these and other developments, it is becoming increasingly important to understand the role of the various arms of natural and vaccine-induced immunity in controlling influenza, and in driving viral evolution. By aiming to link these critical host mechanisms to important phenomena on the population level, mathematical models, such as the one presented here, can be valuable in casting light on the potential impact of new and emerging vaccines.

## Supporting Information

S1 Appendix Supporting Technical Information. (DOCX)
