# Supplementary material for: Universal or Specific? A Modeling-Based Comparison of Broad-Spectrum Influenza Vaccines against Conventional, Strain-Matched Vaccines
Source: PLoS Comput Biol. 2016 Dec 15;12(12):e1005204. doi: 10.1371/journal.pcbi.1005204 (PMC5157952; doi:10.1371/journal.pcbi.1005204)
Supplement: S1 Appendix — (DOCX) [file pcbi.1005204.s001.docx]

**Universal or specific? A modeling-based comparison of broad-spectrum influenza vaccines against conventional, strain-matched vaccines**

**Supporting Technical Information (Appendix S1)**

**A. Flows between immune states in the population**

**Figure A. Schematic summary of the transitions between different immune states in the model.** Here, ‘strain-matched’ immunity is evaluated in the context of a given, circulating strain. ‘Infection-induced immunity’ follows from simulating an influenza season (the ‘epidemic component’ of the model), while ‘viral immune escape’ follows from a model of evolution and immune selection between seasons (the ‘interepidemic’ component). The model also incorporates two types of vaccination program: a ‘conventional’ program that uses current, trivalent inactivated (strain-matched) vaccines, and a ‘universal’ programme, that uses new, universal influenza vaccines. An additional transition, not shown for clarity, is that of population turnover, whereby a fraction *μ* each year go from each of the immune states to the state with no effective immunity.

*The approach*

We concentrate on T-cell vaccines, using ‘cross-protective immunity’ as a shorthand for T-cell immunity that is not specific to HA strains. In the context of a given, circulating HA strain, we distinguish four different types of immune status, as in Fig.A above: (i) those having no effective immunity (W); (ii) those having cross-protective immunity but no HA-specific immunity (X); (iii) those having HA-specific immunity, and no cross-protective immunity (Y); (iv) those having both (Z).

We simulate a series of seasonal epidemics over several years, under a given coverage of conventional and universal vaccination. To model the waning of cross-protective immunity we assume, for simplicity, that each year a proportion $\sigma$ of individuals lose their cross-protective immunity. The loss of strain-matched immunity requires a little extra formulation to keep track of immunizing strains (whether by infection or conventional vaccination), and their relation to circulating strains. To do so, it is convenient to distinguish states in the interepidemic period before and after a seasonal epidemic. We use lowercase and uppercase symbols respectively.

For example, we write *w*_i_ for the proportion of the population that, in the interepidemic period immediately *before* epidemic *i*, lacked any immunity to the virus circulating in season *i*, whether strain-specific or cross-protective immunity. Similarly we write *W*_i_ for this proportion immediately following epidemic *i*. Likewise we write *x_i_* and *X*_i_ for the proportions of the population, respectively before and after epidemic *i*, having cross-protective but no strain-specific immunity.

For the states *Y* and *Z*, which carry HA-specific immunity to the circulating strain, we introduce an additional index *j*, to label the season when HA-specific immunity was acquired. Thus, *y*_ij_ is the proportion of the population that, in the interepidemic period before epidemic *i*, was last immunized by HA-strain *j* and still has HA immunity to this strain but no cross-protective immunity. Similarly we write *Y*_ij_ for this proportion immediately following epidemic *i*. We define *z*_ij_, *Z*_ij_ in the same manner.

The simulation begins with a ‘founding’ virus introduced into a fully naïve population, causing an epidemic labeled as *i* = 0. Thus we have *X*_00_ = 0; *Y*_00_ = 0 and *Z*_00_ as the initial epidemic size. The following steps then iterate from one season to the next.

**1. Interepidemic period separating epidemics *i* and *i* + 1: antigenic drift, and loss of immunity**

*Selection of an immune escape variant*

Let *d*_j_ be the antigenic distance of the virus in season *j*, relative to the founding virus (taking *d*_0_ = 0). For season *i* + 1 we consider a candidate virus with antigenic distance $\delta$from the founding virus, and assuming for simplicity that $\delta$ > *d_i_* (i.e. that antigenic evolution never reverses direction along the assumed 1-dimensional antigenic axis). As a function of $\delta$, the proportion of the population that is susceptible to this candidate virus is:

$$P\left( \delta\right)= W_{i}+\sum_{j\leq i} Y_{i,j}\left[ 1-\exp\left( -\left( \delta-d_{j} \right) \right) \right];$$

$$P^{(cp)}\left( \delta\right)= X_{i}+\sum_{j\leq i} Z_{i,j}\left[ 1-\exp(-(\delta-d_{j})) \right]$$

where *P*^(cp)^, *P* are respectively the susceptible proportions with and without cross-protective immunity. As described in the main text, we therefore select *d*_i+1_ as the value of $\delta$ maximizing the frequency-weighted immune escape, $\left[ P(\delta)+P^{(cp)}(\delta) \right]exp(-k\delta)$.

*Implementing population turnover, waning immunity and viral immune escape*

Immediately prior to epidemic *i* + 1, we then have, for the proportion of the population without effective immunity:

$$w_{i+1}=\mu+\left(1-\mu\right)\left\{W_{i}+X_{i}\sigma+\sum_{j} \left[Y_{ij}f\left(d_{i+1}-d_{j}\right)+Z_{ij}\sigma f\left(d_{i+1}-d_{j}\right)\right]\right\}$$

Here the term in *μ* represents births in the population, and 1 – *μ* captures those individuals who were present during epidemic *i* and are still surviving. The terms in *f* represent the loss of HA-specific immunity as a result of viral immune escape from season *i* to season *i*+1 (see Eq.2 in the main text), and the terms in *σ* represent the waning of cross-protective immunity.

Likewise, for those having cross-protective but not strain-specific immunity:

$$x_{i+1}=\left( 1-\mu\right)\left( 1-\sigma\right)\left[ X_{i}+\sum_{j} f\left( d_{i+1}-d_{j} \right) Z_{i,j} \right]$$

And, for the remaining immune states:

$$\begin{matrix} y_{i+1,j}=(1-\mu)\left[1-f\left(d_{i+1}-d_{j}\right)\right]\left(Y_{ij}+\sigma Z_{ij}\right) \\ z_{i+1,j}=(1-\mu)(1-\sigma)\left[1-f\left(d_{i+1}-d_{j}\right)\right]Z_{ij} \end{matrix}$$

**2. Epidemic period *i + 1: Vaccination and epidemic spread***

*Implementing vaccination*

We assume that any vaccination is completed before the epidemic begins, thus determining the initial conditions for the epidemic as follows. Suppose a proportion *V_C_* of the population receives effective conventional vaccination, and a proportion *V_U_* receives universal vaccination, at random.

Further, we assume that conventional vaccination has efficacy $\epsilon_{C}$, modeled here as the proportion of individuals receiving vaccination who successfully acquire vaccine-induced immunity (the remainder are assumed to remain with whatever susceptibility status they had prior to vaccination). Likewise we assume that universal vaccination has efficacy $\epsilon_{U}$, modeled here by assuming that any vaccinated individuals who become infected have an infectiousness that is diminished by a factor $1-\epsilon_{U}$ (with $\epsilon_{U}=1$ thus corresponding to a fully transmission-blocking vaccine).

The term $\epsilon_{U}$enters the model through the governing equations, as described in the main text, while the term $\epsilon_{C}$ enters as described below.

Following vaccination, and before the start of the epidemic, the susceptible proportions of the population are:

$\begin{matrix} S\left(0\right) =\left(1-\varepsilon_{C}V_{C}\right)\left(1-V_{U}\right)w_{i+1} \\ S^{(cp)}\left(0\right) =\left(1-\varepsilon_{C}V_{C}\right)\left(V_{U}w _{i+1}+x_{i+1}\right) \end{matrix}$,

defining a disease-free equilibrium under random vaccination coverage. Taking the initial conditions as a perturbation to this equilibrium, we solve Eqs. (1) in the main text to find the epidemic size *E,* as a proportion of the population. We therefore have, for the immune states following epidemic *i* + 1:

$$\begin{matrix} W_{i+1}=\left(1-\varepsilon_{C}V_{C}\right)\left(1-V_{U}\right)\left(1-E\right)w_{i+1} \\ X_{i+1}=\left(1-\varepsilon_{C}V_{C}\right)V_{U}\left(1-E\right)w_{i+1}+\left(1-\varepsilon_{C}V_{C}\right)\left(1-E\right)x_{i+1} \\ Y_{i+1,j}=\left\{\begin{matrix} \varepsilon_{C}V_{C}\left(1-V_{U}\right)\left(1-E\right)w_{i+1}+\varepsilon_{C}V_{C}\left(1-V_{U}\right)\sum_{k} y_{i+1,k} & j=i+1 \\ \left(1-\varepsilon_{C}V_{C}\right)\left(1-V_{U}\right)y_{i+1,j} & \text{otherwise} \end{matrix}\right. \\ Z_{i+1,j}=\left\{\begin{matrix} \left[E+\varepsilon_{C}V_{C}V_{U}\left(1-E\right)\right]w_{i+1}+\left[E+\varepsilon_{C}V_{C}\left(1-E\right)\right]x_{i+1}+\varepsilon_{C}V_{C}V_{U}\sum_{k} y_{i+1,k}+\varepsilon_{C}V_{C}\sum_{k} z_{i+1,k} & j=i+1 \\ \left(1-\varepsilon_{C}V_{C}\right)V_{U}y_{i+1,j}+\left(1-\varepsilon_{C}V_{C}\right)z_{i+1,j} & \text{otherwise} \end{matrix}\right. \end{matrix}$$

We then simulate the interepidemic period following epidemic *i* + 1, as described in section 1 above.

**B. A model with ‘leaky’ strain-matched immunity**

In the model presented above we assumed HA-immunity to be ‘all-or-nothing’, that is that a certain proportion of individuals have perfectly neutralizing immunity, while others are fully susceptible. Here we present an alternative model formulation, where HA immunity effects a reduction in susceptibility, in a continuous way depending on the antigenic distance between immunizing and circulating strains. We show that the model, although somewhat more complex than that presented in the main text, yields qualitatively the same behaviour.

In the course of epidemic *i*, we denote by *U*_j_ the proportion of the population that was previously immunized with strain j < i, and remains uninfected by the currently circulating strain *i*. We note that the analogous variable in the ‘default’ model in the main text is *S*: here, the shift in notation serves to emphasise that, under leaky immunity, susceptibility is now subject to variation. In particular, those in compartment *U*_j_ have susceptibility reduced by a factor $g{}_{j}=1-\exp\left[ -\left( d_{i}-d_{j} \right) \right]$.

Further, and similarly to the ‘default’ model, we define *I*_j_ as the proportion infected, and *R*^(cp)^ as the proportion recovered in the current season. As in the main text, recovered individuals necessarily have cross-protective immunity, having acquired natural infection in the current season. The recovered compartment also does not distinguish by *j*, as any prior strain-matched immunity is replaced by immunity to strain *i*.

Thus in epidemic *i*, we have governing equations:

$$\begin{matrix} \frac{dU_{j}}{dt}=-g_{j}U_{j}\lambda& \frac{dI_{j}}{dt}=g_{j}U_{j}\lambda-\gamma I_{j} \\ \frac{dU_{j}^{(cp)}}{dt}=-g_{j}U_{j}^{(cp)}\lambda& \frac{dI_{j}^{\left( cp \right)}}{dt}=g_{j}U_{j}^{(cp)}\lambda-\gamma I_{j}^{(cp)} \\ \frac{dR^{\left( cp \right)}}{dt}=\sum_{j} \gamma(I_{j}+I_{j}^{(cp)}) & \end{matrix}$$

where the force-of-infection is given by $\lambda= \beta\sum_{j} [I_{j}+\left( 1-c \right)I_{j}^{\left( cp \right)}]$. By integrating these equations analytically, it is then straightforward to show that epidemic *i* will infect a proportion *p*_j_ of group *j,* such that

$$\ln(1-p_{j})=g_{j}\frac{\beta}{\gamma}\sum_{k} p_{k}\left[ U{}_{k}(0)+\left( 1-c \right)U_{k}^{\left( cp \right)}(0) \right]$$

Solving this equation for *p*_j_, we define the epidemic size *E*_j_ in group *j* as

$$E_{j}=p_{j}\left[ U{}_{j}\left( 0 \right)+\left( 1-c \right)U_{j}^{\left( cp \right)}\left( 0 \right) \right]$$

Figure B below shows results from this modified model, showing similar overall results to those illustrated in Figure 3: for a given coverage, and matched by efficacy, conventional vaccines allow for greater seasonal epidemic sizes than universal vaccines (left panel); universal vaccines dampen antigenic evolution while conventional vaccines tend to have the opposite effect (center panel); and, in the event of a pandemic, a population utilizing conventional vaccines for routine seasonal vaccination tends to allow greater pandemic sizes than one utilizing universal vaccines (right panel).

**Figure B**. Illustrative results under a model assuming ‘leaky’ strain-specific immunity, under which immune individuals have reduced susceptibility, depending on the antigenic distance between immunizing and circulating strains. Model results show qualitatively similar behaviour to those demonstrated in Fig.3 in the main text. In pandemic sizes (rightmost panel), the plateau represents the case of a fully naïve population (without cross-protective or strain-specific immunity), a scenario in which the pandemic size is limited only by *R*_0_.

**Figure C. Sensitivity analysis of relative efficiency in controlling seasonal epidemics, with fixed half-life.** This figure is plotted in the same way as Fig.5 in the main text, but holding constant the half-life *h* of cross-protective immunity at 3 years. As a result, relative efficiencies in controlling seasonal epidemics are strictly lower than 1.

**C. Matlab code used for simulations in the main text**

%% Name: epidemic_simulation_R2.m

%% Author: Rahul Subramanian

% Summary: Simulates a given number of epidemics (numepis), with pandemics

% occuring initially and every 25 years thereafter. Note that the

% simulation begins with index 2.

% Dependencies: find_E_comp_newton_method.m

% Execution: [equilibrium_epidemic_size, pandemic_size, equilibrium_antigenic_evolution] = epidemic_simulation(numepis,imm_eff_cp, q, t,vac_eff_cp, halfLife, k,CPVacFreq)

function [equilibrium_epidemic_size, pandemic_size, equilibrium_antigenic_evolution] = epidemic_simulation_R2(numepis,imm_eff_cp, conventional_vac_coverage, universal_vac_coverage,vac_eff_universal, halfLife, k,CPVacFreq, Beta, mu)

%Beta = 2.0; %Baseline transmission number, equivalent to baseline reproductive number (R_0) since recovery rate gamma = 1.

%mu = 0.02; %Population turnover rate

%% Pre-epidemic notation:

y = zeros(numepis+1, numepis+1); %% Proportion of population without any cross-protective immunity

% in the ith season who last obtained HA-specific immunity in in the jth season having taken into account any turnover that have may have occured

% between the two seasons.

w = zeros(numepis+1,1);%%Proportion of population without any cross-protective

% or HA-specific immunity as of the ith season.

x = zeros(numepis+1); %Proporition of indiviudals

% with cross-protective immunity but no HA-specific

% immunity prior to year i

z = zeros(numepis+1, numepis+1); % Proportion of individuals

% with both HA-specific immunity (acquired during year

% j) as well as cross-protective immunity prior to year

% i

%% Post-epidemic notation:

Y = zeros(numepis+1, numepis+1);

W = zeros(numepis+1,1);

X = zeros(numepis+1,1);

Z = zeros(numepis+1, numepis+1);

% Intially, the whole population lacks either HA-specific

% or cross-protective immunity

gamma = 1; %Recovery Rate

thres = 1e6; %Accuracy threshold for antigenic evolution calculations

delta = linspace(0, 1, thres); %%vector of values for antigenic distance from previous strain

d = zeros(1,numepis+1); %% antigenic distance taken at each epidemic cycle

%% Record-Keeping Vectors

Epi_size_sto = zeros(1, numepis+1); %% Records the size of each epidemic

ant_evol_sto = zeros(1, numepis+1); %% Records the amount of antigenic evolution

%% Preceding each epidemic

time = 1; %Assume one-year since last adjustment for half-life

%%(Across all time cohorts regardless of time of last

%%infection).

sigma = (1-((.5)^(time/halfLife)));

tsp = 0; %Time since last pandemic

tsp_list = zeros(1,numepis);

vac_eff_conventional = imm_eff_cp;

Epsilon_c = vac_eff_conventional;

V_c = conventional_vac_coverage;

for i = 1:numepis

tsp_list(i) = tsp;

%Determine if cross-protective vaccination occurs

%in this cycle

CPVac = mod(i,CPVacFreq);

if(CPVac == 0)

V_u = universal_vac_coverage*vac_eff_universal*imm_eff_cp;

else

V_u = 0;

end

if(tsp == 0)

%%Pandemic Case:

%Pandemic Adjustment

if( i == 1)

w(2) = 1;

else

%%Implementing Population Turnover, Waning Immunity,

%%and Viral Immune Escape

w(i+1) = mu + (1-mu)*[W(i)+sum(Y(i,:)) + [sigma]*[X(i) + sum(Z(i,:))]];

x(i+1) = (1-mu)*[1-sigma]*[X(i) + sum(Z(i,:))];

y(i+1,:) = zeros(1,numepis+1);

z(i+1,:)= zeros(1,numepis+1);

sum_total = W(i) + X(i)+ sum(Y(i,:)) + sum(Z(i,:));

dif = abs(sum_total-1);

assert(dif<1e-14, 'Dif Observed %f at %f',dif, i );

sum_total = w(i+1) + x(i+1)+ sum(y(i+1,:)) + sum(z(i+1,:));

dif = abs(sum_total-1);

assert(dif<1e-14, 'Dif Observed %f at %f',dif, i );

end

%% Selection of an Immune Escape Variant

P = w(i+1);

P_cp = x(i+1);

d(i+1) = d(i);

%% Calculate size of susceptible population given

% that vaccination already occured

S_0 = w(i+1)*(1-V_u);

S_cp_0 = x(i+1)+w(i+1)*V_u;

R_eff = Beta*[S_0 + (1-imm_eff_cp)*S_cp_0];

E_comp = find_E_comp_newton_method(R_eff);

E = 1-E_comp;

Epi_size_sto(i+1)= E*(S_0+S_cp_0);

%% Update Immune States After Vaccination and Epidemic

W(i+1) = (1-V_u)*(1-E)*w(i+1);

X(i+1) = V_u*(1-E)*w(i+1)+(1-E)*x(i+1);

Y(i+1,i+1) = 0;

Y(i+1,1:i) = zeros(1,i);

Z(i+1,i+1) = [E]*(w(i+1)) + E*x(i+1);

Z(i+1,1:i) = zeros(1,i);

sum_total = W(i+1) + X(i+1)+ sum(Y(i+1,:)) + sum(Z(i+1,:));

dif = abs(sum_total-1);

assert(dif<1e-14, 'Dif Observed %f at %f',sum_total, i );

panIndex = i;

else

%% Seasonal Epidemic

%% Selection of an Immune Escape Variant

P = W(i);

P_cp = X(i);

for j = 1:i

P = P + (Y(i,j).*(1-exp(-(delta + d(i)-d(j)))));

P_cp = P_cp + (Z(i,j).*(1-exp(-(delta + d(i)-d(j)))));

end

frequency_weighted_immune_escape = (P + (1-imm_eff_cp)*P_cp).*exp(-k*delta);

%frequency_weighted_immune_escape = (P + P_cp).*exp(-k*delta);

strain_index = find(frequency_weighted_immune_escape == max(frequency_weighted_immune_escape) );

strain_index = strain_index(end);

ant_evol_sto(i+1) = delta(strain_index);

if(i>1)

d(i+1) = d(i) + ant_evol_sto(i+1);

else

d(i+1) = ant_evol_sto(i+1);

end

%%Implementing Population Turnover, Waning Immunity,

%%and Viral Immune Escape

w(i+1) = mu + (1-mu)*[W(i)+sum(Y(i,1:i).*(1-exp(-(d(i+1)-d(1:i)))))+X(i)*sigma+sum(Z(i,1:i).*(1-exp(-(d(i+1)-d(1:i)))))*sigma];

y(i+1,1:i) = (1-mu)*[[Y(i,1:i)+Z(i,1:i)*sigma].*[1-(1-exp(-(d(i+1)-d(1:i))))]];

x(i+1) = (1-mu)*(1-sigma)*[X(i) + sum(Z(i,1:i).*(1-exp(-(d(i+1)-d(1:i)))))];

z(i+1,1:i) = (1-mu)*(1-sigma)*[Z(i,1:i)].*[1-(1-exp(-(d(i+1)-d(1:i))))];

sum_total = W(i) + X(i)+ sum(Y(i,:)) + sum(Z(i,:));

dif = abs(sum_total-1);

assert(dif<1e-14, 'Dif Observed %f at %f',sum_total, i );

sum_total = w(i+1) + x(i+1)+ sum(y(i+1,:)) + sum(z(i+1,:));

dif = abs(sum_total-1);

assert(dif<1e-14, 'Dif Observed %f at %f',dif, i );

%% Calculate size of susceptible population given

% that vaccination already occured

S_0 = w(i+1)*(1-V_c*Epsilon_c)*(1-V_u);

S_cp_0 = (1-V_c*Epsilon_c)*V_u*w(i+1)+ (1-V_c*Epsilon_c)*x(i+1);

R_eff = Beta*[S_0 + (1-imm_eff_cp)*S_cp_0];

E_comp = find_E_comp_newton_method(R_eff);

E = 1-E_comp;

Epi_size_sto(i+1)= E*(S_0+S_cp_0);

sum_total = w(i+1) + x(i+1)+ sum(y(i+1,1:i)) + sum(z(i+1,1:i));

dif = abs(sum_total-1);

assert(dif<1e-14, 'Dif Observed %f at %f',dif, i );

%% Update Immune States After Vaccination and Epidemic

W(i+1) = (1-V_c*Epsilon_c)*(1-V_u)*(1-E)*w(i+1);

X(i+1) = (1-V_c*Epsilon_c)*V_u*(1-E)*w(i+1)+(1-V_c*Epsilon_c)*(1-E)*x(i+1);

Y(i+1,i+1) = V_c*Epsilon_c*(1-V_u)*w(i+1) + V_c*Epsilon_c*(1-V_u)*sum(y(i+1,1:i));

Y(i+1,1:i) = (1-V_c*Epsilon_c)*(1-V_u)*y(i+1,1:i);

Z(i+1,i+1) = [E*(1-V_c*Epsilon_c) + V_c*Epsilon_c*V_u]*[w(i+1)]+V_c*Epsilon_c*V_u*sum(y(i+1,1:i)) +[E*(1-V_c*Epsilon_c)+V_c*Epsilon_c]*[x(i+1)] +V_c*Epsilon_c*sum(z(i+1,1:i));

Z(i+1,1:i) = (1-V_c*Epsilon_c)*V_u*y(i+1,1:i) + (1-V_c*Epsilon_c)*z(i+1,1:i);

sum_total = W(i+1) + X(i+1)+ sum(Y(i+1,:)) + sum(Z(i+1,:));

dif = abs(sum_total-1);

assert(dif<1e-14, 'Dif Observed %f at %f',sum_total, i );

end

%% Pandemic checks

if(tsp == 25)

tsp = 0; %Next epidemic will be a pandemic

else

tsp = tsp + 1; %Increment time since last pandemic

end

end

if(panIndex >1)

equilibrium_epidemic_size = mean(Epi_size_sto((panIndex+1)-9 : (panIndex+1)-1));

equilibrium_antigenic_evolution= mean(ant_evol_sto((panIndex+1)-9 : (panIndex+1)-1));

else

equilibrium_epidemic_size = mean(Epi_size_sto(end-9:end));

equilibrium_antigenic_evolution = mean(ant_evol_sto(end-9:end));

end

pandemic_size = Epi_size_sto(panIndex+1);

end
